# Risk of adverse outcomes following urinary tract infection in older people with renal impairment: Retrospective cohort study using linked health record data

**DOI:** 10.1371/journal.pmed.1002652

**Published:** 2018-09-10

**Authors:** Haroon Ahmed, Daniel Farewell, Nick A. Francis, Shantini Paranjothy, Christopher C. Butler

**Affiliations:** 1 Division of Population Medicine, Cardiff University School of Medicine, Cardiff, United Kingdom; 2 Nuffield Department of Primary Care Health Sciences, University of Oxford, Oxford, United Kingdom; Royal Derby Hospital, UNITED KINGDOM

## Abstract

**Background:**

Few studies have investigated the risk of adverse outcomes in older people with renal impairment presenting to primary care with a urinary tract infection (UTI). The aim of this study was to determine the risk of adverse outcomes in patients aged ≥65 years presenting to primary care with a UTI, by estimated glomerular filtration rate (eGFR) and empirical prescription of nitrofurantoin versus trimethoprim.

**Methods and findings:**

This was a retrospective cohort study using linked health record data from 795,484 patients from 393 general practices in England, who were aged ≥65 years between 2010 and 2016. Patients were entered into the cohort if they presented with a UTI and had a creatinine measurement in the 24 months prior to presentation. We calculated an eGFR to estimate risk of adverse outcomes by renal function, and propensity-score matched patients with eGFRs <60 mL/minute/1.73 m^2^ to estimate risk of adverse outcomes between those prescribed trimethoprim and nitrofurantoin. Outcomes were 14-day risk of reconsultation for urinary symptoms and same-day antibiotic prescription (proxy for treatment nonresponse), hospitalisation for UTI, sepsis, or acute kidney injury (AKI), and 28-day risk of death. Of 123,607 eligible patients with a UTI, we calculated an eGFR for 116,945 (95%). Median age was 76 (IQR, 70–83) years and 32,428 (28%) were male. Compared to an eGFR of >60 mL/minute/1.73 m^2^, patients with an eGFR of <60 mL/minute/1.73 m^2^ had greater odds of hospitalisation for UTI (adjusted odds ratios [ORs] ranged from 1.14 [95% confidence interval (CI) 1.01–1.28, *p* = 0.028], for eGFRs of 45–59, to 1.68 [95% CI 1.01–2.82, *p* < 0.001] for eGFRs <15) and AKI (adjusted ORs ranged from 1.57 [95% CI 1.29–1.91, *p* < 0.001], for eGFRs of 45–59, to 4.53 [95% CI 2.52–8.17, *p* < 0.001] for eGFRs <15). Compared to an eGFR of >60 mL/minute/1.73 m^2^, patients with an eGFR <45 had significantly greater odds of hospitalisation for sepsis, and those with an eGFR <30 had significantly greater odds of death. Compared to trimethoprim, nitrofurantoin prescribing was associated with lower odds of hospitalisation for AKI (ORs ranged from 0.62 [95% CI 0.40–0.94, *p* = 0.025], for eGFRs of 45–59, to 0.45 [95% CI 0.25–0.81, *p* = 0.008] for eGFRs <30). Nitrofurantoin was not associated with greater odds of any adverse outcome. Our study lacked data on urine microbiology and antibiotic-related adverse events. Despite our design, residual confounding may still have affected some of our findings.

**Conclusions:**

Older patients with renal impairment presenting to primary care with a UTI had an increased risk of UTI-related hospitalisation and death, suggesting a need for interventions that reduce the risk of these adverse outcomes. Nitrofurantoin prescribing was not associated with an increased risk of adverse outcomes in patients with an eGFR <60 mL/minute/1.73 m^2^ and could be used more widely in this population.

## Abstract 2

In this retrospective study, Haroon Ahmed and colleagues investigate associated risks between common urinary tract infection and impaired kidney function in older adults, as well as outcomes related to nitrofurantoin prescription.

## Author summary

**Why was this study done?:**

- It is not known if older adults with impaired kidney function are at increased risk of hospitalisation or death following a urinary tract infection (UTI).
- Nitrofurantoin is an antibiotic used to treat UTI but is not recommended in people with impaired kidney function. However, the evidence supporting this recommendation is limited.

**What did the researchers do and find?:**

- This study used linked health record data from general practices and hospitals in England and estimated risk of hospitalisation and death for older adults with impaired kidney function presenting to primary care with a suspected UTI.
- Older adults with impaired kidney function had greater risk of a UTI-related hospitalisation and death in the 14–28 days following a UTI.
- Older adults with impaired kidney function who were treated with nitrofurantoin were not at greater risk of an adverse outcome and were less likely to experience a hospital admission for worsening kidney function.

**What do these findings mean?:**

- There is a need for strategies that prevent UTIs and reduce the risk of UTI-related hospitalisations and death in older adults with impaired kidney function.
- Nitrofurantoin was not associated with worse outcomes and could be used more widely in this population.

## Introduction

The Kidney Disease Improving Global Outcomes working group defines degrees of renal impairment using the glomerular filtration rate (GFR) [1]. GFRs <60 mL/minute/1.73 m^2^ are split into four groups and reflect worsening renal function, from mild impairment to renal failure. Around 6% of adults in the United Kingdom have an estimated glomerular filtration rate (eGFR) of <60 mL/minute/1.73 m^2^ [2]. This increases with age to around 20% of those aged ≥65. There is increasing evidence of an association between renal impairment and infection [3–6]. Oxidative stress in renal impairment disrupts the function of inflammatory cytokines and may impair immune response during an infection [7]. In more severe renal impairment, uremic toxins impair the function of T-lymphocyte and antigen-presenting cells, which play important roles in cellular and humoral immunity [8]. Despite the high prevalence in older adults, and the association with infection, few studies have investigated outcomes following an infectious illness in older people with renal impairment.

A cohort study in UK primary care showed that around 20% of adults aged 65 and over had at least one urinary tract infection (UTI) over a median follow-up of 5 years [9]. Most adults presenting to primary care with symptoms and signs of a UTI receive empirical antibiotics at the same consultation, without knowledge of microbiological findings or antibiotic susceptibilities [10]. Nitrofurantoin and trimethoprim (alone or with sulfamethoxazole) are the two most commonly prescribed antibiotics for empirical treatment of UTIs and are recommended by clinical guidelines in the UK, United States, and Europe [11,12]. Nitrofurantoin use was initially limited to those with an eGFR ≥60 mL/minute/1.73 m^2^, because of concerns about poorer efficacy in patients with lower eGFRs. In 2014, a review of the evidence [13] and a retrospective cohort study [14] prompted the UK Medicines and Healthcare products Regulation Authority to lower the threshold for nitrofurantoin use to an eGFR ≥45 mL/minute/1.73 m^2^. However, outcomes following empirical nitrofurantoin prescribing in older adults with a UTI and an eGFR <60 mL/minute/1.73 m^2^ are yet to be fully evaluated. There are also concerns about trimethoprim use in older adults with renal impairment, with increasing evidence of an association with hyperkalaemia and sudden death, especially when prescribed to patients already taking angiotensin-converting enzyme inhibitors, angiotensin-II receptor blockers, or potassium-sparing diuretics [15–19].

We used data from anonymised linked health records to estimate the risk of adverse outcomes in older patients with renal impairment empirically treated for suspected UTI in primary care. We firstly compared outcomes by eGFR to understand whether severity of renal impairment was associated with risk of adverse outcome following a UTI. This would help identify which patients would most benefit from interventions that improved prevention and/or treatment of UTI. We also compared outcomes for older patients with an eGFR <60 mL/minute/1.73 m^2^ who were prescribed empirical nitrofurantoin versus empirical trimethoprim to inform prescribing decisions and explore if nitrofurantoin prescribing is safe in patients with renal impairment.

## Methods

### Data source

We used the Clinical Practice Research Datalink (CPRD), an electronic database of anonymised primary care records covering 11.3 million patients from 674 general practices across the UK [20]. Approximately 7% of the UK population are included and patients are broadly representative of the wider UK population in terms of age, sex, and ethnicity. The CPRD holds data on demographics, clinical encounters, diagnoses (coded using Read codes), drug prescriptions, laboratory tests, and referrals to specialists. Data are available once they have met a series of quality checks on completeness and reliability and the CPRD deems them to be of the standard required for research purposes. Linked hospital and death registration data are available for patients from approximately 50% of contributing English practices. Hospital diagnoses and causes of death are recorded using the International Classification of Diseases-10th Revision (ICD-10).

The CPRD Independent Scientific Advisory Committee approved the study protocol and analysis plan (protocol number 17_250, S1 Protocol). Further ethical approval was not required as the proposed research was within the remit of the CPRD’s broad National Research Ethics Service approval. We used the Reporting of Studies Conducted using Observational Routinely-collected Health Data (RECORD) statement and checklist (S1 RECORD Checklist) to guide study reporting [21].

### Design and participants

This was a retrospective cohort study using linked health record data. Patients were eligible for inclusion if, between 1 January 2010 and 31 December 2016, their data were of the quality required by CPRD, they were ≥65 years old, and eligible for data linkage. Only patients registered with practices that had consented to data linkage would have linked hospital and death registry data. We excluded patients if they were temporary residents (i.e., they registered with the practice for an acute problem but this was not their normal ‘long-term’ practice and thus medical record data would be limited) or had periods during their registration with the practice for which CPRD was unable to collect data, potentially leading to incomplete exposure/event capture. We identified eligible patients with a Read code indicating an incident primary care presentation with a suspected UTI (codes available in S1 Appendix), a prescription code indicating same-day empirical prescribing of a relevant antibiotic, and a creatinine record in the preceding 24 months. We defined ‘incident’ as a presentation without a previous consultation with a UTI-related Read code, or trimethoprim or nitrofurantoin prescription in the preceding 90 days. We used the first incident episode during each patient’s follow-up period. We excluded UTI episodes with a hospital discharge in the preceding 14 days to exclude hospital-acquired infections.

### Exposures

We used the most recent serum creatinine value recorded in the 24 months preceding the incident UTI and data for patient age, gender, and ethnicity to calculate an eGFR as per the Modification of Diet in Renal Disease (MDRD) Study equation [22]. We categorised eGFRs as ≥60 mL/minute/1.73 m^2^, 45–59 mL/minute/1.73 m^2^, 30–44 mL/minute/1.73 m^2^, 15–29 mL/minute/1.73 m^2^, and <15 mL/minute/1.73 m^2^. These categories are similar to those used by the UK National Institute of Health and Care Excellence to categorise the stages of chronic kidney disease, except we combined eGFRs of 60–89 mL/minute/1.73 m^2^ with those ≥90 mL/minute/1.73 m^2^. This was because eGFRs ≥60 mL/minute/1.73 m^2^ without additional evidence of kidney damage are clinically regarded as normal, and previous research found no difference in infection incidence or outcome between these two groups [5]. We used those with an eGFR ≥60 mL/minute/1.73 m^2^ as the reference and compared rates of adverse outcomes against the four other eGFR categories. We used the recorded empirical antibiotic prescription as the exposure variable to compare risk of adverse outcomes between patients with eGFRs <60 mL/minute/1.73 m^2^ prescribed trimethoprim versus nitrofurantoin.

### Outcomes

We assessed the impact of our stated exposures on the following adverse outcomes for patients empirically treated in primary care for an incident suspected UTI:

1. Reconsultation for urinary symptoms and a same-day antibiotic prescription within 14 days following the incident UTI, as a proxy for treatment nonresponse, ascertained through Read and prescription codes recorded in primary care records.
2. Hospitalisation for UTI, sepsis, or acute kidney injury (AKI) within 14 days following the incident UTI ascertained from ICD-10 codes recorded in linked hospital admission data for the first episode of a hospital admission, i.e., the episode most likely responsible for the admission.
3. Death within 28 days following the incident UTI using linked death registration data.

We also initially planned to include hospitalisation for pyelonephritis as an outcome. However, our exploratory work showed that pyelonephritis was rarely coded in hospital records (only 8 events in total) and thus was unlikely to be a reliable outcome for use with these data.

### Statistical analyses

We used primary care demographic and clinical codes to describe baseline characteristics for patients by exposure status. To assess the impact of eGFR, we compared rates of each outcome in those with an eGFR ≥60 mL/minute/1.73 m^2^ to those in each category related to an eGFR <60 mL/minute/1.73 m^2^, and used logistic regression to estimate odds ratios (ORs) and 95% confidence intervals (CIs). We adjusted for potential confounders of the association between renal impairment and outcome, including age; Index of Multiple Deprivation score quintile; Charlson comorbidity score [23]; the presence or absence of a record indicating diabetes, dementia, coronary heart disease, stroke, cancer, and heart failure; and polypharmacy. We inferred the presence of polypharmacy if the patient’s record showed repeated monthly prescribing of ≥5 medications in the year prior to the incident UTI. We also adjusted for the choice and duration of antibiotic therapy used to treat the incident UTI.

To assess the impact of empirical trimethoprim versus nitrofurantoin prescribing, we used a range of demographic and clinical variables to match patients on their propensity to receive a trimethoprim prescription. These included the confounders listed above and presence or absence of a record indicating urinary incontinence or long-term catheterisation, and long-term prescribing of angiotensin-converting enzyme inhibitors, angiotensin-II receptor blockers, or potassium-sparing diuretics. We used nearest neighbour matching with no replacement and matched each patient with a nitrofurantoin prescription to three patients with trimethoprim prescriptions. We assessed balance in measured baseline covariates between matched groups by visually inspecting jitter plots and histograms of covariate distribution before and after matching, and by calculating standardised mean differences for covariates between groups. We regarded standardised mean differences of <0.1 as reflecting adequate balance [24,25]. We used mixed effects logistic regression to account for clustering by general practice and calculated ORs and 95% CIs for each outcome.

All statistical tests were two-sided, with *p* < 0.05 considered statistically significant but an effect size of 10% considered clinically significant. *p*-values were derived using two-tailed Wald tests. Analyses were conducted in R version 3.2.1.

## Results

**Fig 1:**
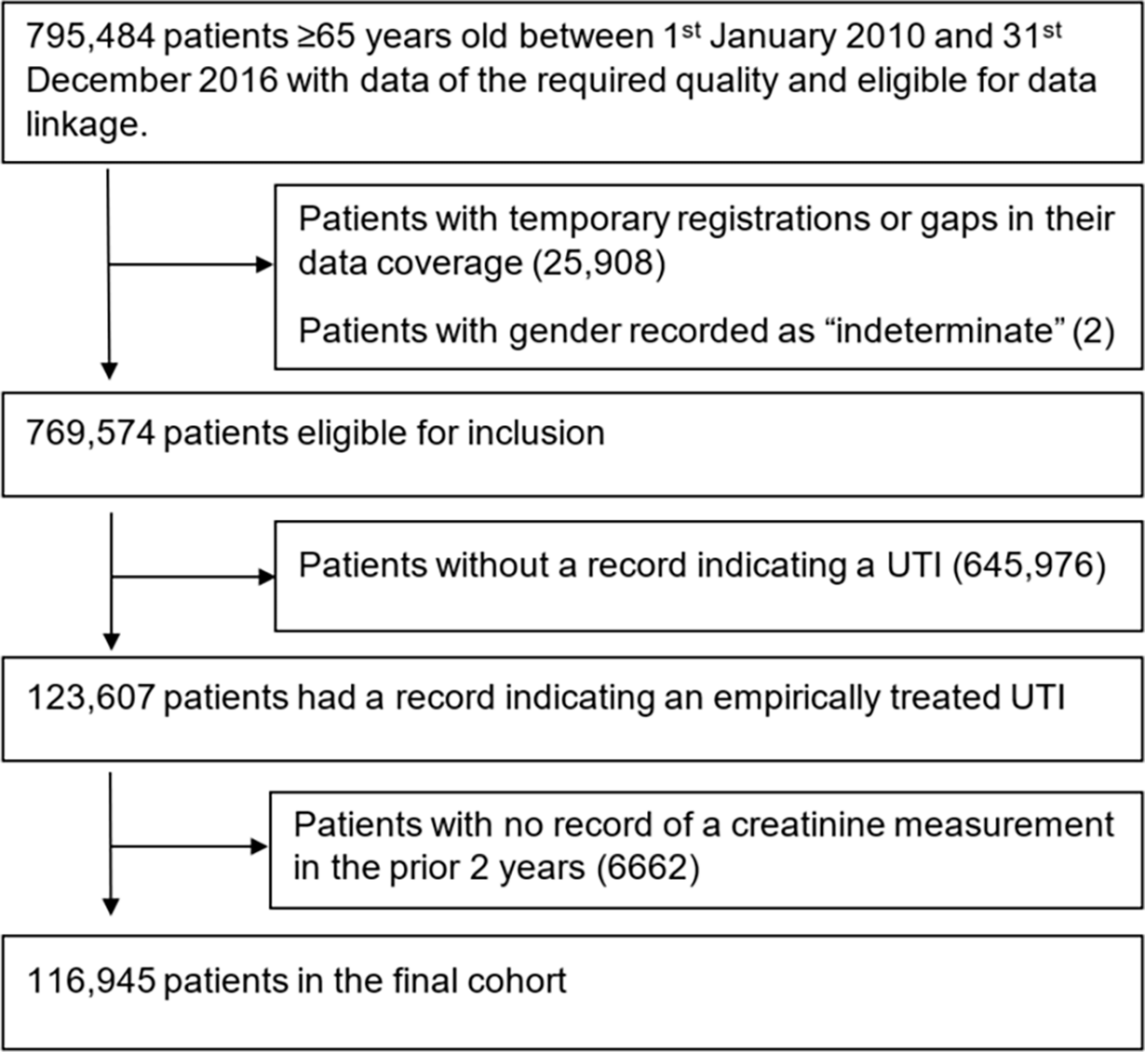
Flow of patients from initial identification in the database through to final cohort. UTI, urinary tract infection.

From a cohort of 795,484 patients aged 65 and over, we identified 123,607 with an incident UTI empirically treated with a relevant antibiotic (**Fig 1**). Of these, 116,945 (95%) patients had a creatinine measurement recorded in the 24 months prior to the incident UTI. In this final cohort, 32,428 (28%) were male and the median age at the time of incident UTI was 76 years (IQR 70–83). Almost one third of creatinine measurements were in the 90 days prior to the incident UTI. Median duration between most recent creatinine and UTI was 169 days (IQR 65–285). Using the MDRD study equation, 76,112 (65.1%) of patients were assigned an eGFR ≥60, 26,970 (23.1%) an eGFR of 45–59, 10,854 (9.3%) an eGFR of 30–44, 2,667 (2.3%) an eGFR of 15–29, and 342 (0.3%) an eGFR of <15. Baseline characteristics showed that patients with lower eGFRs had a relatively greater number of comorbidities and comprised greater proportions of patients with polypharmacy (**Table 1**). Trimethoprim was the most commonly prescribed empirical antibiotic across all eGFR groups. Nitrofurantoin was the second most common except in patients with an eGFR <15 mL/minute/1.73 m^2^.

**Table 1:** Baseline characteristics of included patients according to eGFR category.

Characteristics	eGFR ≥60	eGFR 45–59	eGFR 30–44	eGFR 15–29	eGFR <15
***N***	76,112 (65.1)	26,970 (23.1)	10,854 (9.3)	2,667 (2.3)	342 (0.3)
**Men**	21,816 (28.7)	6,674 (24.7)	2,894 (26.7)	880 (33.0)	164 (48.0)
**Mean (SD) age**	75.2 (7.9)	79.1 (8.3)	82.5 (8.0)	83.2 (8.0)	79 (8.2)
**Prescribed antibiotic**					
Amoxicillin Cefalexin Ciprofloxacin Co-amoxiclav Nitrofurantoin Trimethoprim	3,370 (4.4) 4,168 (5.5) 2,344 (3.1) 3,170 (4.2) 16,719 (22.0) 46,341 (60.9)	1,367 (5.1) 1,749 (6.5) 811 (3.0) 1,227 (4.5) 5,237 (19.4) 16,579 (61.5)	680 (6.3) 917 (8.4) 388 (3.6) 613 (5.6) 1,815 (16.7) 6,441 (59.3)	289 (10.8) 319 (12.0) 148 (5.5) 208 (7.8) 391 (14.7) 1,312 (49.2)	45 (13.2) 47 (13.7) 26 (7.6) 44 (12.9) 41 (12.0) 139 (40.6)
**Index of multiple deprivation decile**					
1 or 2 (least deprived) 3 or 4 5 or 6 7 or 8 9 or 10 (most deprived)	19,939 (26.2) 18,413 (24.2) 16,606 (21.8) 12,283 (16.1) 8,871 (11.7)	6,401 (23.7) 6,524 (24.2) 5,859 (21.7) 4,626 (17.2) 3,560 (13.2)	2,292 (21.1) 2,526 (23.3) 2,443 (22.5) 1,918 (17.7) 1,675 (15.4)	560 (21.0) 619 (23.2) 615 (23.1) 477 (17.9) 396 (14.8)	65 (19.0) 77 (22.5) 85 (24.9) 45 (13.2) 70 (20.5)
**Housebound**	2,261 (3.0)	1,201 (4.5)	866 (8.0)	267 (10.0)	34 (9.9)
**Respiratory disease**	15,853 (20.8)	5,521 (20.5)	2,208 (20.3)	538 (20.2)	46 (13.5)
**Cardiac failure**	2,187 (2.9)	1,733 (6.4)	1,367 (12.6)	499 (18.7)	56 (16.4)
**Dementia**	3,724 (4.9)	2,024 (7.5)	1,129 (10.4)	265 (9.9)	21 (6.1)
**Peripheral vascular disease**	2,903 (3.8)	1,502 (5.6)	953 (8.8)	319 (12.0)	43 (12.6)
**Rheumatoid arthritis**	2,140 (2.8)	803 (3.0)	339 (3.1)	97 (3.6)	9 (2.6)
**Cancer**	11,291 (14.8)	4,211 (15.6)	1,891 (17.4)	498 (18.7)	82 (24.0)
**Stroke**	6,714 (8.8)	3,123 (11.6)	1,695 (15.6)	454 (17.0)	67 (19.6)
**Diabetes**	11,956 (15.7)	5,103 (18.9)	2,863 (26.4)	961 (36.0)	112 (32.7)
**Liver disease**	445 (0.6)	165 (0.6)	69 (0.6)	11 (0.4)	3 (0.9)
**Ischaemic heart disease**	11,611 (15.3)	5,814 (21.6)	3,118 (28.7)	878 (32.9)	114 (33.3)
**Urinary catheter**	2,360 (3.1)	854 (3.2)	504 (4.6)	193 (7.2)	39 (11.4)
**Urinary incontinence**	10,966 (14.4)	4,089 (15.2)	1,702 (15.7)	398 (14.9)	41 (12.0)
**Polypharmacy**	24,478 (32.2)	11,419 (42.3)	6,371 (58.7)	1,797 (67.4)	237 (69.3)
**Potassium-sparing diuretic**	1,470 (1.9)	946 (3.5)	732 (6.7)	158 (5.9)	4 (1.2)
**Angiotensin-converting enzyme inhibitor**	16,430 (21.6)	7,586 (28.1)	3,446 (31.7)	718 (26.9)	46 (13.5)
**Angiotensin-II receptor antagonist**	8,195 (10.8)	3,933 (14.6)	1,885 (17.4)	453 (17.0)	42 (12.3)
**Charlson score**					
0 1 2 3 4 5 ≥6	30,663 (40.3) 18,770 (24.7) 12,973 (17.0) 7,394 (9.7) 3,219 (4.2) 1,636 (2.1) 1,457 (1.9)	6,131 (22.7) 4,100 (15.2) 6,309 (23.4) 4,666 (17.3) 2,725 (10.1) 1,661 (6.2) 1,378 (5.1)	879 (8.1) 824 (7.6) 2,621 (24.1) 2,424 (22.3) 1,728 (15.9) 1,148 (10.6) 1,230 (11.3)	104 (3.9) 116 (4.3) 504 (18.9) 577 (21.6) 481 (18.0) 401 (15.0) 484 (18.1)	6 (1.8) 12 (3.5) 81 (23.7) 64 (18.7) 66 (19.3) 45 (13.2) 68 (19.9)

Numbers are values (%) unless otherwise stated.

Abbreviation: eGFR, estimated glomerular filtration rate.

### Outcomes according to calculated eGFR

There were 7,203 reconsultations with urinary symptoms resulting in another antibiotic prescription within 14 days of the incident UTIs, equating to about 6% of the cohort. The odds of reconsulting and receiving another antibiotic prescription were no different between those with an eGFR ≥60 mL/minute/1.73 m^2^ and those with eGFRs <60mL/minute/1.73 m^2^ (Table 2).

**Table 2:** Adjusted ORs and 95% CIs for each outcome by eGFR category.

Reconsultation and re-prescription within 14 days	Number of UTIs	Number (%) of events	Crude OR	Adjusted* OR (95% CI)	*p*-value
eGFR ≥60	76,112	4,852 (6.4)	1	1	
eGFR 45–59	26,970	1,563 (5.8)	0.90	0.97 (0.91–1.03)	0.328
eGFR 30–44	10,854	626 (5.8)	0.90	1.03 (0.94–1.14)	0.511
eGFR 15–29	2,667	148 (5.5)	0.86	1.02 (0.86–1.22)	0.804
eGFR <15	342	14 (4.1)	0.63	0.72 (0.42–1.23)	0.224
**Hospitalised for UTI within 14 days**					
eGFR ≥60	76,112	1,003 (1.3)	1	1	
eGFR 45–59	26,970	526 (2.0)	1.49	1.14 (1.01–1.28)	0.028
eGFR 30–44	10,854	317 (2.9)	2.25	1.25 (1.08–1.44)	0.003
eGFR 15–29	2,667	129 (4.8)	3.81	1.76 (1.43–2.16)	<0.001
eGFR <15	342	16 (4.7)	3.68	1.68 (1.01–2.82)	<0.001
**Hospitalised for sepsis within 14 days**					
eGFR ≥60	76,112	77 (0.1)	1	1	
eGFR 45–59	26,970	46 (0.2)	1.69	1.36 (0.92–2.01)	0.119
eGFR 30–44	10,854	32 (0.3)	2.92	1.70 (1.06–2.72)	0.027
eGFR 15–29	2,667	17 (0.6)	6.33	2.72 (1.50–4.94)	<0.001
eGFR <15	342	4 (1.2)	11.69	4.24 (1.48–11.23)	0.007
**Hospitalised for AKI within 14 days**					
eGFR ≥60	76,112	280 (0.4)	1	1	
eGFR 45–59	26,970	204 (0.8)	2.06	1.57 (1.29–1.91)	<0.001
eGFR 30–44	10,854	231 (2.1)	5.89	3.21 (2.61–3.94)	<0.001
eGFR 15–29	2,667	137 (5.1)	14.67	6.70 (5.24–8.55)	<0.001
eGFR <15	342	13 (3.8)	10.70	4.53 (2.52–8.17)	<0.001
**Death within 28 days**					
eGFR ≥60	76,112	588 (0.8)	1	1	
eGFR 45–59	26,970	285 (1.1)	1.37	0.92 (0.79–1.07)	0.275
eGFR 30–44	10,854	201 (1.9)	2.42	1.05 (0.87–1.26)	0.598
eGFR 15–29	2,667	99 (3.7)	4.95	1.63 (1.27–2.10)	<0.001
eGFR <15	342	19 (5.6)	7.56	2.37 (1.44–3.89)	<0.001

*ORs adjusted for age, gender, Index of Multiple Deprivation score quintile, Charlson comorbidity score, being housebound, respiratory disease, peripheral vascular disease, liver disease, rheumatoid arthritis, diabetes, dementia, coronary heart disease, stroke, cancer, heart failure, polypharmacy, long-term prescribing of angiotensin-converting enzyme inhibitors, angiotensin-II receptor blockers, or potassium-sparing diuretics, urinary catheter, urinary incontinence, and choice and duration of antibiotic therapy.

Abbreviations: AKI, acute kidney injury; CI, confidence interval; eGFR, estimated glomerular filtration rate; OR, odds ratio; UTI, urinary tract infection.

There were 1,991 hospitalisations for UTI (1.7% of the cohort), 176 for sepsis (0.2% of the cohort), and 865 for AKI (0.7% of the cohort) within 14 days of the incident UTIs. Compared to those with an eGFR ≥60 mL/minute/1.73 m^2^, odds of hospitalisation for UTI increased in those with eGFRs of 45–59 (adjusted OR 1.14, 95% CI 1.01–1.28), 30–44 (adjusted OR 1.25, 95% CI 1.08–1.44), 15–29 (adjusted OR 1.76, 95% CI 1.43–2.16), and <15 (adjusted OR 1.68, 95% CI 1.01–2.82). Odds of hospitalisation for sepsis were no different in those with an eGFR of 45–59 but were significantly higher in those with eGFRs of 30–44 (adjusted OR 1.70, 95% CI 1.06–2.72), 15–29 (adjusted OR 2.72, 95% CI 1.50–4.94), and <15 (adjusted OR 4.24, 95% CI 1.48–11.23). The risk of hospitalisation for AKI increased in a graded manner relative to renal function, with adjusted ORs of 1.57 (95% CI 1.29–1.91), 3.21 (95% CI 2.61–3.94), 6.70 (95% CI 5.24–8.55), and 4.53 (95% CI 2.52–8.17) for eGFRs of 45–59, 30–44, 15–29, and <15 mL/minute/1.73 m^2^, respectively.

There were 1,162 deaths in the 28 days following the incident UTIs, equating to about 1% of the cohort. Compared to those with an eGFR ≥60 mL/minute/1.73 m^2^, the odds of death were no different in those with an eGFR ≥30 mL/minute/1.73 m^2^, 63% higher in those with an eGFR of 15–29 (adjusted OR 1.63, 95% CI 1.27–2.10), and over 2-fold higher in those with an eGFR <15 (adjusted OR 2.37, 95% CI 1.44–3.89).

### Trimethoprim versus nitrofurantoin in those with an eGFR <60 mL/minute/1.73 m^2^

Of the 40,833 patients with an eGFR <60 mL/minute/1.73 m^2^, 24,471 (60%) were prescribed trimethoprim and 7,484 (18%) were prescribed nitrofurantoin. We matched 20,948 patients with an eGFR of 45–60 (15,711 prescribed trimethoprim, 5,237 prescribed nitrofurantoin), 7,260 with an eGFR of 30–44 (5,445 prescribed trimethoprim, 1,815 prescribed nitrofurantoin), and 1,728 with an eGFR <30 (1,296 prescribed trimethoprim, 432 prescribed nitrofurantoin). Inspection of jitter plots and histograms suggested matching had improved balance of covariates across trimethoprim versus nitrofurantoin groups. Standardised mean differences were all less than 0.1 (Table 3).

**Table 3:** Balance of baseline characteristics across trimethoprim and nitrofurantoin groups following propensity score matching for patients with renal impairment.

**eGFR 45–60 mL/minute/1.73 m**^**2**^	**Trimethoprim**	**Nitrofurantoin**	**Standardised mean difference**
***N***	15,711	5,237	
**Men**	3,421 (21.8)	1,120 (21.4)	0.01
**Mean (SD) age**	78.9 (8.3)	78.8 (8.2)	−0.02
**Index of multiple deprivation decile**			
1 or 2 (least deprived) 3 or 4 5 or 6 7 or 8 9 or 10 (most deprived)	3,746 (23.8) 3,807 (24.2) 3,488 (22.2) 2,711 (17.3) 1,959 (12.5)	1,305 (24.9) 1,257 (24.0) 1,088 (20.8) 867 (16.6) 720 (13.7)	0.00
**Housebound**	637 (4.1)	206 (3.9)	−0.01
**Respiratory disease**	3,173 (20.2)	1,115 (21.3)	0.00
**Cardiac failure**	952 (6.1)	317 (6.1)	0.00
**Dementia**	1,111 (7.1)	361 (6.9)	−0.01
**Cancer**	2,389 (15.2)	853 (16.3)	0.03
**Stroke**	1,768 (11.3)	611 (11.7)	0.01
**Diabetes**	2,890 (18.4)	956 (18.3)	0.00
**Ischaemic heart disease**	3,244 (20.6)	1,098 (21)	0.01
**Urinary catheter**	406 (2.6)	176 (3.4)	0.00
**Urinary incontinence**	2,337 (14.9)	883 (16.9)	0.05
**Polypharmacy**	6,497 (41.4)	2,262 (43.2)	0.04
**Potassium-sparing diuretic**	551 (3.5)	197 (3.8)	0.01
**Angiotensin-converting enzyme inhibitor**	4,364 (27.8)	1,437 (27.4)	−0.01
**Angiotensin-II receptor antagonist**	2,294 (14.6)	786 (15)	0.01
**Charlson score**			
0 1 2 3 4 5 ≥6	3,802 (24.2) 2,426 (15.4) 3,669 (23.4) 2,632 (16.8) 1,561 (9.9) 891 (5.7) 730 (4.6)	1,212 (23.1) 814 (15.5) 1,256 (24) 904 (17.3) 490 (9.4) 313 (6.0) 248 (4.7)	0.02
**eGFR 30–44 mL/minute/1.73 m**^**2**^	**Trimethoprim**	**Nitrofurantoin**	**Standardised mean difference**
***N***	5,445	1,815	
**Men**	1,201 (22.1)	414 (22.8)	−0.02
**Mean (SD) age**	82.3 (7.9)	82.2 (8.0)	−0.01
**Index of multiple deprivation decile**			
1 or 2 (least deprived) 3 or 4 5 or 6 7 or 8 9 or 10 (most deprived)	1,162 (21.3) 1,219 (22.4) 1,229 (22.6) 986 (18.1) 849 (15.6)	360 (19.8) 466 (25.7) 394 (21.7) 301 (16.6) 294 (16.2)	0.00
**Housebound**	475 (8.7)	167 (9.2)	0.02
**Respiratory disease**	1,148 (21.1)	384 (21.2)	0.00
**Cardiac failure**	657 (12.1)	226 (12.5)	0.01
**Dementia**	605 (11.1)	201 (11.1)	0.00
**Cancer**	924 (17)	314 (17.3)	0.01
**Stroke**	880 (16.2)	304 (16.7)	0.02
**Diabetes**	1,493 (27.4)	527 (29)	0.04
**Ischaemic heart disease**	1,517 (27.9)	503 (27.7)	0.00
**Urinary catheter**	240 (4.4)	112 (6.2)	0.07
**Urinary incontinence**	882 (16.2)	302 (16.6)	0.01
**Polypharmacy**	3,302 (60.6)	1,117 (61.5)	0.02
**Potassium-sparing diuretic**	391 (7.2)	137 (7.5)	0.01
**Angiotensin-converting enzyme inhibitor**	1,758 (32.3)	593 (32.7)	0.01
**Angiotensin-II receptor antagonist**	941 (17.3)	307 (16.9)	−0.01
**Charlson score**			
0 1 2 3 4 5 ≥6	441 (8.1) 441 (8.1) 1,283 (23.6) 1,237 (22.7) 894 (16.4) 550 (10.1) 599 (11.0)	147 (8.1) 144 (7.9) 442 (24.4) 381 (21.0) 273 (15.0) 213 (11.7) 215 (11.8)	0.02
**eGFR <30 mL/minute/1.73 m**^**2**^	**Trimethoprim**	**Nitrofurantoin**	**Standardised mean difference**
***N***	1,296	432	
**Men**	339 (26.2)	113 (26.2)	0.00
**Mean (SD) age**	83.4 (8.2)	83.4 (8.0)	0.00
**Index of multiple deprivation decile**			
1 or 2 (least deprived) 3 or 4 5 or 6 7 or 8 9 or 10 (most deprived)	281 (21.7) 297 (22.9) 286 (22.1) 231 (17.8) 201 (15.5)	93 (21.5) 95 (22.0) 100 (23.1) 79 (18.3) 65 (15.0)	0.02
**Housebound**	131 (10.1)	42 (9.7)	−0.01
**Respiratory disease**	250 (19.3)	86 (19.9)	0.02
**Cardiac failure**	224 (17.3)	71 (16.4)	−0.02
**Dementia**	132 (10.2)	42 (9.7)	−0.02
**Cancer**	229 (17.7)	74 (17.1)	−0.01
**Stroke**	215 (16.6)	71 (16.4)	0.00
**Diabetes**	451 (34.8)	155 (35.9)	0.02
**Ischaemic heart disease**	406 (31.3)	132 (30.6)	−0.02
**Urinary catheter**	93 (7.2)	29 (6.7)	−0.02
**Urinary incontinence**	193 (14.9)	63 (14.6)	−0.01
**Polypharmacy**	877 (67.7)	294 (68.1)	0.01
**Potassium-sparing diuretic**	76 (5.9)	23 (5.3)	−0.02
**Angiotensin-converting enzyme inhibitor**	340 (26.2)	112 (25.9)	−0.01
**Angiotensin-II receptor antagonist**	220 (17.0)	79 (18.3)	0.03
**Charlson score**			
0 1 2 3 4 5 ≥6	51 (3.9) 57 (4.4) 291 (22.5) 276 (21.3) 229 (17.7) 179 (13.8) 213 (16.4)	21 (4.9) 21 (4.9) 92 (21.3) 93 (21.5) 79 (18.3) 56 (13.0) 70 (16.2)	−0.03

Numbers are values (%) unless otherwise stated.

Abbreviation: eGFR, estimated glomerular filtration rate.

Empirical nitrofurantoin prescribing was associated with lower odds of hospitalisation for AKI across all eGFR groups (eGFR 45–59: OR 0.62, 95% CI 0.40–0.94; eGFR 30–44: OR 0.47, 95% CI 0.30–0.73; eGFR <30: OR 0.45, 95% CI 0.25–0.81) (Table 4). Nitrofurantoin was also associated with lower odds of reconsultation and re-prescription in patients with eGFRs of 45–59 (OR 0.74, 95% CI 0.61–0.91) and lower odds of death in patients with eGFRs of 30–44 (OR 0.61, 95% CI 0.39–0.95). There were no other statistically significant differences between empirical trimethoprim versus nitrofurantoin prescribing. Importantly, we did not detect any increase in odds of adverse outcomes in patients prescribed nitrofurantoin.

**Table 4:** ORs and 95% CIs for each outcome in propensity-score* matched trimethoprim versus nitrofurantoin groups, across three eGFR categories.

**eGFR 45–59**	**Trimethoprim group, *n* = 15,711**	**Nitrofurantoin group, *n* = 5,237**	**OR (95% CI)**	***p*-value**
	**Number (%) of events**	**Number (%) of events**		
Death within 28 days	159 (1.0)	50 (1.0)	0.94 (0.69–1.30)	0.718
Reconsultation and re-prescription within 14 days	942 (6.0)	290 (5.5)	0.74 (0.61–0.91)	0.004
Hospitalised for UTI within 14 days	288 (1.8)	105 (2.0)	1.09 (0.74–1.61)	0.648
Hospitalised for sepsis within 14 days	25 (0.2)	6 (0.72)	0.72 (0.30–1.76)	0.470
Hospitalised for AKI within 14 days	126 (0.8)	26 (0.5)	0.62 (0.40–0.94)	0.025
**eGFR 30–44**	**Trimethoprim group, *n* = 5,445**	**Nitrofurantoin group, *n* = 1,815**		
	**Number (%) of events**	**Number (%) of events**	**OR (95% CI)**	***p*-value**
Death within 28 days	113 (2.1)	23 (1.3)	0.61 (0.39–0.95)	<0.001
Reconsultation and re-prescription within 14 days	318 (5.8)	117 (6.4)	0.98 (0.71–1.33)	0.874
Hospitalised for UTI within 14 days	168 (3.1)	57 (3.1)	0.80 (0.44–1.47)	0.482
Hospitalised for sepsis within 14 days	14 (0.3)	2 (0.1)	0.43 (0.10–1.88)	0.262
Hospitalised for AKI within 14 days	146 (2.7)	23 (1.3)	0.47 (0.30–0.73)	<0.001
**eGFR <30**	**Trimethoprim group, *n* = 1,296**	**Nitrofurantoin group, *n* = 432**		
	**Number (%) of events**	**Number (%) of events**	**OR (95% CI)**	***p*-value**
Death within 28 days	49 (3.8)	18 (4.2)	1.11 (0.64–1.93)	0.713
Reconsultation and re-prescription within 14 days	74 (5.7)	29 (6.7)	1.19 (0.76–1.85)	0.446
Hospitalised for UTI within 14 days	73 (5.6)	23 (5.3)	0.94 (0.58–1.53)	0.808
Hospitalised for sepsis within 14 days	8 (0.6)	2 (0.5)	0.75 (0.16–3.54)	0.715
Hospitalised for AKI within 14 days	84 (6.5)	13 (3.0)	0.45 (0.25–0.81)	0.008

*The following baseline variables were used in the propensity-score model: age, gender, Index of Multiple Deprivation score quintile, Charlson comorbidity score, being housebound, the presence or absence of respiratory disease, peripheral vascular disease, liver disease, rheumatoid arthritis, diabetes, dementia, coronary heart disease, stroke, cancer, heart failure, urinary catheter, urinary incontinence, polypharmacy, long-term prescribing of angiotensin-converting enzyme inhibitors, angiotensin-II receptor blockers, or potassium-sparing diuretics.

Abbreviations: AKI, acute kidney injury; CI, confidence interval; eGFR, estimated glomerular filtration rate; OR, odds ratio; UTI, urinary tract infection.

### Sensitivity analyses

We restricted our eGFR and outcomes analysis to patients with a creatinine measured in the 90 days prior to the incident UTI, to increase the likelihood that the calculated eGFR reflected their current renal function. Results were consistent with our main analysis and most of the statistically significant ORs increased in magnitude (S1 Table). We also combined the hospitalisation and death outcomes in our trimethoprim versus nitrofurantoin analysis to increase statistical power to detect these adverse outcomes (S2 Table). Findings were consistent with our main analysis.

## Discussion

Our results show that compared to an eGFR of >60 mL/minute/1.73 m^2^, older patients with an eGFR of <60 mL/minute/1.73 m^2^ who were empirically treated for suspected UTI in primary care had greater odds of hospitalisation for UTI and AKI, those with an eGFR <45 had greater odds of hospitalisation for sepsis, and those with an eGFR <30 had greater odds of death. The magnitude of each association generally increased relative to the severity of the renal impairment. We also showed that, compared to trimethoprim, nitrofurantoin was associated with reduced odds of hospitalisation for AKI across all eGFR groups and was not associated with an increased risk of any adverse event evaluated in our study.

### Results in context

Previous research focussed on the risk of infection-related hospitalisation in adults with renal impairment and showed a greater risk of hospitalisation for pneumonia, UTI, bacteraemia, and cellulitis in those with eGFRs <60 mL/minute/1.73 m^2^ [4–6]. Previous studies also showed a greater risk of death following an infection-related hospitalisation in patients with renal impairment [3–5] but provided little information on health service contact prior to the adverse outcome, thus limiting interpretation about possible opportunities to intervene. Our study shows an increased risk of infection-related hospitalisation and death in older adults with renal impairment, following infection-related presentation and treatment in primary care. We also show increased odds of AKI hospitalisation in those with lower initial eGFRs, previously only investigated in a small cohort (*n* = 790) of patients hospitalised with UTI, who likely had more severe infection on initial presentation [26]. To the best of our knowledge, this is the first study to investigate the impact of eGFR on odds of reconsultation and re-prescription following an infection-related illness. We found no difference in the odds of this outcome across the different eGFR groups, suggesting that the increased odds of UTI, sepsis, and death were less likely to be due to treatment nonresponse and more likely to be related to other patient or renal factors.

Trimethoprim (with or without sulfamethoxazole) prescribing is associated with an increased risk of hyperkalaemia, AKI, and death, compared to amoxicillin [16–19]. Amoxicillin accounts for only about 5% of prescribing for UTI in the UK [9] and thus is a less helpful comparator for clinical decision-making. Furthermore, these studies did not investigate associations by degree of renal impairment, providing little information to guide prescribing in this population. Two studies assessed trimethoprim and nitrofurantoin prescribing in patients with renal impairment. The first compared treatment failure rates in women with UTI prescribed nitrofurantoin according to renal function and found no difference across the eGFR groups [14]. This study lacked a comparator group prescribed an alternative antibiotic, which makes it difficult to interpret their findings. The second compared outcomes in older women with a median eGFR of 38 mL/minute/1.73 m^2^, prescribed either nitrofurantoin or trimethoprim, and found no difference in risk of treatment failure or UTI hospitalisation [27]. We compared nitrofurantoin with trimethoprim across three eGFR groups and found that nitrofurantoin was associated with lower odds of reconsultation and re-prescription in patients with eGFRs of 45–59. This could be explained by recent data showing that 34% of community-acquired *Escherichia coli* UTIs in England are resistant to trimethoprim, compared to only 2.7% resistant to nitrofurantoin [28]. We did not find statistically significant differences between reconsultation and re-prescription rates in people with eGFRs <45. This could be due to less statistical power, as nitrofurantoin use was less common in these patients because of the advice to use with care in patients with eGFRs of 30–44 and to avoid in eGFRs <30. It may also be due to the possibility that nitrofurantoin efficacy was reduced in those with lower eGFRs but was offset by the high rates of trimethoprim resistance and thus resulted in apparent similar rates of reconsultation and re-prescription. Our finding that nitrofurantoin was associated with a reduced risk of death in those with moderate renal impairment is consistent with previously reported estimates in studies that compared nitrofurantoin with amoxicillin in the general population [18,19]. We also found a previously unreported lower risk of AKI associated with nitrofurantoin use across all three eGFR groups of our cohort.

### Strengths and weaknesses of this study

We used data from a general practice database that is broadly representative of the UK population, increasing the generalisability of our findings. This is the largest cohort study to investigate the impact of eGFR on infection-related outcomes, with a sample size >4 times larger than the previously largest study [4]. Cohort entry was dependent on presentation and empirical treatment of UTI in primary care, and thus reduced indication bias. We adjusted for the presence/absence of more comorbidities than previous studies, increasing the likelihood of an independent association between eGFR and adverse outcomes. This is the first study to investigate trimethoprim versus nitrofurantoin prescribing in renal impairment, using clinically relevant eGFR groups analogous to stages of chronic kidney disease and without excluding men. We also reduced indication bias by matching patients on their propensity to receive trimethoprim, and achieving adequate balance of covariates across the two groups.

Our study has important limitations. We attempted to capture patients presenting with UTI but had no microbiological data to support this. However, whilst a limitation, this is also more representative of clinical practice. We were unable to investigate pulmonary/hepatic toxicity related to nitrofurantoin use because of the lack of reliable codes, and differential use of these codes by clinicians. However, two systematic reviews have shown that these toxicities are rare with short-term use [29,30]. We relied on a creatinine measurement from the 24 months prior to the UTI to estimate an eGFR, but this may not fully represent patients’ current renal function. Finally, despite our design, differential coding, indication bias, and residual confounding may still have affected our findings.

### Clinical implications

Around 20% of adults aged ≥65 present to primary care at least once with a UTI [9], and around 20% have renal impairment [2] and thus are at greater risk of an adverse outcome. The initial primary care visit presents a potential opportunity to address this increased risk. However, recommended interventions such as stopping co-prescribed angiotensin-converting enzyme inhibitors or angiotensin-II receptor blockers have not been evaluated in the primary care setting [31]. Therefore, there is a need for research that evaluates primary care–based interventions that may prevent adverse outcomes, including AKI, in patients with renal impairment and community-acquired infection. The absolute risks of hospitalisation and death are low. Therefore, interventions may be best targeted at those at highest risk—i.e., those with more severe renal impairment.

Current guidelines and the British National Formulary limit nitrofurantoin use to those with an eGFR >45 mL/minute/1.73 m^2^, although short courses can be used with care in those with eGFRs >30 mL/minute/1.73 m^2^ [11]. We found no evidence to support this limitation and actually found nitrofurantoin to be associated with a reduced risk of AKI, compared to trimethoprim. Research suggesting poorer nitrofurantoin efficacy in patients with renal impairment assessed urinary nitrofurantoin excretion, not clinical outcomes, and was restricted to small samples [13]. Our findings, combined with increasing rates of bacterial resistance to trimethoprim and the importance of avoiding broader spectrum agents, support wider use of nitrofurantoin for older patients with low eGFRs.

### Conclusion

Our findings show that older patients with renal impairment presenting to primary care with a UTI are at greater risk of adverse outcomes independent of other comorbidities and of prescribed empirical antibiotic treatment. Despite documented concerns, we found no increased risk of adverse outcomes in patients with an eGFR <60 mL/minute/1.73 m^2^ prescribed nitrofurantoin and support its wider use in selected patients with moderate-severe renal impairment.

**Disclaimer:** The views expressed in this publication are those of the authors and not necessarily those of the NIHR, NHS Wales, HCRW, or the Welsh Government.

## Supporting information

S1 RECORD Checklist RECORD statement. RECORD, reporting of studies conducted using observational routinely-collected health data.
(DOCX)

S1 Appendix Identifying clinically diagnosed UTI using read and ICD-10 codes. UTI, urinary tract infection.
(DOCX)

S1 Table Adjusted odds ratios and 95% confidence intervals for each outcome by eGFR category restricted to the 37,379 patients with a creatinine measurement in the 90 days prior to the UTI event. eGFR, estimated glomerular filtration rate.
(DOCX)

S2 Table Adjusted odds ratios and 95% confidence intervals for a combined ‘hospitalisation or death’ outcome in matched trimethoprim versus nitrofurantoin groups, across three eGFR categories. eGFR, estimated glomerular filtration rate.
(DOCX)

S1 Protocol Study protocol. (DOCX)

## Abbreviations

AKI: acute kidney injury
CI: confidence interval
CPRD: Clinical Practice Research Datalink
eGFR: estimated glomerular filtration rate
GFR: glomerular filtration rate
MDRD: Modification of Diet in Renal Disease
OR: odds ratio
RECORD: Reporting of Studies Conducted using Observational Routinely-collected Health Data
UTI: urinary tract infection
