# Supplementary material for: Risk of adverse outcomes following urinary tract infection in older people with renal impairment: Retrospective cohort study using linked health record data
Source: PLoS Med. 2018 Sep 10;15(9):e1002652. doi: 10.1371/journal.pmed.1002652 (PMC6130857; doi:10.1371/journal.pmed.1002652)
Supplement: S1 Table — eGFR, estimated glomerular filtration rate. (DOCX) [file pmed.1002652.s003.docx]

S1 Table: Adjusted odds ratios and 95% confidence intervals for each outcome by eGFR category restricted to 37,379 patients with a creatinine measurement in the 90 days prior to the UTI event.

| **Reconsultation and antibiotic prescription within 14 days** | **adjusted OR (95% CI)** | **p-value** |
| --- | --- | --- |
| eGFR ≥60 | 1 | 1 |
| eGFR 45-59 | 1.02 (0.91-1.13) | 0.782 |
| eGFR 30-44 | 1.12 (0.96-1.30) | 0.138 |
| eGFR 15-29 | 1.02 (0.80-1.30) | 0.879 |
| eGFR <15 | 0.54 (0.24-1.24) | 0.146 |
|  |  |  |
| **Hospitalised for UTI within 14 days** |  |  |
| eGFR ≥60 | 1 | 1 |
| eGFR 45-59 | 1.22 (1.01-1.47) | 0.036 |
| eGFR 30-44 | 1.41 (1.13-1.76) | 0.002 |
| eGFR 15-29 | 2.18 (1.65-2.87) | <0.001 |
| eGFR <15 | 1.95 (1.00-3.79) | 0.050 |
|  |  |  |
| **Hospitalised for sepsis within 14 days** |  |  |
| eGFR ≥60 | 1 | 1 |
| eGFR 45-59 | 1.48 (0.72-3.01) | 0.285 |
| eGFR 30-44 | 1.96 (0.87-4.38) | 0.102 |
| eGFR 15-29 | 5.94 (2.62-13.47) | <0.001 |
| eGFR <15 | 8.03 (2.10-30.71) | 0.002 |
|  |  |  |
| **Hospitalised for AKI within 14 days** |  |  |
| eGFR ≥60 | 1 | 1 |
| eGFR 45-59 | 1.66 (1.20-2.28) | 0.002 |
| eGFR 30-44 | 3.47 (2.52-4.79) | <0.001 |
| eGFR 15-29 | 9.39 (6.65-13.26) | <0.001 |
| eGFR <15 | 7.31 (3.61-14.80) | <0.001 |
|  |  |  |
| **Death within 28 days** |  |  |
| eGFR ≥60 | 1 | 1 |
| eGFR 45-59 | 0.88 (0.68-1.14) | 0.336 |
| eGFR 30-44 | 0.92 (0.68-1.24) | 0.573 |
| eGFR 15-29 | 2.19 (1.57-3.05) | <0.001 |
| eGFR <15 | 3.72 (2.08-6.63) | <0.001 |
