# Supplementary material for: Risk of adverse outcomes following urinary tract infection in older people with renal impairment: Retrospective cohort study using linked health record data
Source: PLoS Med. 2018 Sep 10;15(9):e1002652. doi: 10.1371/journal.pmed.1002652 (PMC6130857; doi:10.1371/journal.pmed.1002652)
Supplement: S2 Table — eGFR, estimated glomerular filtration rate. (DOCX) [file pmed.1002652.s004.docx]

S2 Table: Adjusted odds ratios and 95% confidence intervals for a combined “hospitalisation or death” outcome in matched trimethoprim versus nitrofurantoin groups, across three eGFR categories.

| **eGFR** | **Number (%) of events* in trimethoprim group** | **Number (%) of events* in nitrofurantoin group** | **OR (95% CI)** | **p-value** |
| --- | --- | --- | --- | --- |
| 45-59 | 491 (3.1) | 157 (3.0) | 0.96 (0.80-1.15) | 0.645 |
| 30-44 | 329 (6.0) | 82 (4.5) | 0.48 (0.30-0.77) | 0.002 |
| <30 | 147 (11.0) | 43 (10.0) | 0.86 (0.60-1.24) | 0.425 |

*Events from the following outcomes were combined: Hospitalisation for UTI, sepsis and AKI within 14 days and death within 28 days.
