# Supplementary material for: Risk of adverse outcomes following urinary tract infection in older people with renal impairment: Retrospective cohort study using linked health record data
Source: PLoS Med. 2018 Sep 10;15(9):e1002652. doi: 10.1371/journal.pmed.1002652 (PMC6130857; doi:10.1371/journal.pmed.1002652)
Supplement: S1 Protocol — (DOCX) [file pmed.1002652.s005.docx]

**PROTOCOL INFORMATION REQUIRED**

The following sections below **must** be included in the CPRD ISAC research protocol. Please refer to the guidance on ‘***Contents of CPRD ISAC Research Protocols***’ ([www.cprd.com/isac](http://www.cprd.com/isac)) for more information on how to complete the sections below. Pages should be numbered. All abbreviations must be defined on first use.

| **Applicants must complete all sections listed below**  **Sections which do not apply should be completed as ‘*Not Applicable’*** |
| --- |
| 1. **Study Title^§^**   **^§^***Please note:* *This information will be published on CPRD’s website as part of its transparency policy*  Outcomes following empirical antibiotic treatment of suspected urinary tract infection in older people in primary care. |
| 1. **Lay Summary (Max. 200 words)^§^**   **^§^***Please note:* *This information will be published on CPRD’s website as part of its transparency policy*  Urinary tract infections (UTIs), also known as “bladder infections” or “cystitis”, are a common cause of illness in older people. Older people with symptoms of a UTI usually see their GP and may need antibiotics. Although some GPs wait for results of a urine test (48-72 hours) before prescribing antibiotics, most will prescribe at the initial consultation based on presenting signs and symptoms. Little is known about the rates of adverse outcomes in older people managed in this way, for example, rates receiving an alternative antibiotic because of persisting symptoms, of hospitalisation for serious infection, or of sudden worsening of kidney function. There is a need for research that quantifies the risk of these outcomes for different antibiotics and different durations of treatment, to help GPs make informed decisions around immediate antibiotic treatment of suspected UTI. Immediate prescribing of the most appropriate antibiotic, for the most appropriate duration, is important for antimicrobial stewardship and may reduce risk of adverse outcomes, particularly in high-risk patients, like those with underlying kidney disease. Therefore, this research will assess outcomes for older people prescribed immediate antibiotics for suspected UTI and compare outcomes for different antibiotics, different durations, and in patients with kidney disease. |
| 1. **Technical Summary (Max. 200 words**)**^§^**   **^§^***Please note:* *This information will be published on CPRD’s website as part of its transparency policy*  Urinary tract infections (UTIs) are a common cause of morbidity in older people. Most older people with a suspected UTI consult their GP and may receive a prescription for empirical antibiotics. Little is known about rates of adverse outcomes following this approach, nor about associations between different empirical antibiotic prescribing strategies and outcomes, especially in higher risk groups, e.g., those with impaired renal function. There is also uncertainty around the optimal duration of treatment in this population, with limited evidence for the current practice of three-day therapy in women and seven-day therapy in men. This study will address these evidence gaps using linked CPRD gold, HES admitted patient care, and ONS death registry data. We will compare outcomes for different antibiotic prescribing strategies in patients aged ≥65 empirically treated for suspected UTI in primary care, and estimate risks of adverse outcomes according to renal function. We will explore potential predictors of early serious adverse outcomes. We will use multivariable logistic regression and propensity score-matching methods to estimate risk ratios and adjust for a range of confounding variables. Our research will inform clinical practice by providing evidence for appropriate empirical antibiotic prescribing for older people presenting to primary care with suspected UTI. |
| 1. **Objectives, Specific Aims and Rationale**   The overall objective of this study is to improve current management of suspected UTI in older people in primary care by providing estimates of the comparative effectiveness of different empiric antibiotic prescribing strategies and identifying patient characteristics that increase the odds of a serious adverse outcome.  Specific aims are to:   1. Estimate risk of re-consultation, antibiotic prescribing, hospitalisation and death in a cohort of adults aged ≥65 years empirically treated for UTI in primary care, according to choice and duration of antibiotic therapy, and renal function. 2. Describe causes and predictors of early hospitalisation and death.   Rationale for each aim are discussed in the background section. |
| 1. **Study Background**   UTI is an important cause of morbidity and health service use in older adults. In the UK, general practitioners manage most UTIs with empirical antibiotic treatment, i.e., antibiotics prescribed for a presumed diagnosis of UTI based on clinical presentation, without knowledge of urinary microbiological findings. However, there are several key gaps in the evidence base that need to be addressed to better inform clinical decision making in this area.  Firstly, there are few studies estimating risk of adverse outcomes from empirically treated UTI in a population generalizable to UK primary care. Previous research only describes outcomes in select populations, e.g., older patients with diabetes ^1^ or older US Veterans. ^2^  Secondly, there are few adequately generalizable, current, comparative effectiveness studies of empirical antibiotic therapy for UTI. A recent network meta-analysis showed that 9 of 12 randomised trials excluded patients over 65, and all 12 excluded men. ^3^ Furthermore, most trials compared broad-spectrum antibiotics, and prescribing of these agents in UK primary care is discouraged given their impact on antibiotic resistance. Another meta-analysis of randomised trials of nitrofurantoin also highlighted the lack of older people, and the various biases associated with these trials, most of which were conducted between 1970 and 1990. ^4^ Previous observational studies have generally not examined antibiotics commonly used for UTI in the UK.^5 6^ Therefore, there is a need to compare outcomes across the different antibiotics currently used for UTI treatment in the UK. There are also on-going uncertainties around most appropriate therapy duration in this population. A meta-analysis of six randomised trials showed no difference between clinical outcomes in older women with UTI prescribed 3-6 days of antibiotics versus those prescribed 7-14 days, ^7^ but recent data show continued wide variation in duration of prescribed therapy in UK primary care. ^8^ The uncertainty is greater in older men, with no randomised trial data to support the clinical guideline recommendation of seven-day therapy. ^9^  Thirdly, although nitrofurantoin use for UTI treatment has increased in those with renal impairment, the clinical impact of its use in this population requires further evaluation. The MHRA advice to allow use in renal impairment was based primarily on a systematic review showing limited evidence for previous recommendations to avoid nitrofurantoin in those with an estimated glomerular filtration rate of <60ml/min/1.73m^2^, ^10^ and an observational study of women with UTI showing no difference in treatment failure according to renal function. ^11^ A more recent observational study however, suggests nitrofurantoin is associated with higher rates of treatment failure in those with impaired renal function when compared with other antibiotics. ^12^ Given the mixed evidence and the lack of evidence for men, the use of nitrofurantoin in patients with renal impairment needs further evaluation.  Finally, there is little evidence to help clinicians identify patients with urinary tract symptoms at risk of an early serious adverse outcome. Identifying patient characteristics that increase the odds of a serious adverse event may help to individualise clinical assessment and management of these patients. Describing causes of early hospitalisation or death may provide insights into potentially preventable causes, and help to improve clinical care. |
| 1. **Study Type**   Aim 1 (comparative effectiveness) is hypothesis testing.  Aim 2 (predictors and causes of early adverse outcomes) is hypothesis generating. |
| 1. **Study Design**   Retrospective cohort studies using traditional regression methods and propensity-score matching. |
| 1. **Feasibility counts**   Feasibility counts were undertaken using data from our previous CPRD project (15_027A). We inspected data from 1^st^ January 2013 to 31^st^ December 2013. There were approximately 450,000 patients aged ≥65, from up-to-standard practices, with acceptable data and eligibility for data-linkage. 33,753 (7.5%) had a UTI read code, of whom 33,080 (98%) had a same-day antibiotic prescription.  Table below shows counts for the 6 most commonly prescribed antibiotics, by gender.   \| **Antibiotic** \| \| \| \| \| \| \| \| --- \| --- \| --- \| --- \| --- \| --- \| --- \| \| **Gender** \| **Trimethoprim** \| **Nitrofurantoin** \| **Amoxicillin** \| **Cefalexin** \| **Co-amoxiclav** \| **Ciprofloxacin** \| \| **Male** \| 4316 \| 1607 \| 524 \| 363 \| 545 \| 484 \| \| **Female** \| 14409 \| 6405 \| 1209 \| 1227 \| 871 \| 546 \| |
| 1. **Sample size considerations**   We estimated study power using a conservative difference between proportions approach using the “pwr” package in R version 3.1.1. We plan to use nitrofurantoin as our main comparator. Therefore, using the smallest numbers from the above table for men and ciprofloxacin, 1607 men were prescribed nitrofurantoin and 484 men were prescribed ciprofloxacin, thus giving 90% power to detect a 17% difference in our stated outcomes, at the alpha=0.05 level. We expect our final sample sizes to be greater, given that we will use data from Jan 2010 – Dec 2016, and thus expect to be able to detect smaller differences. We will re-estimate power upon confirmation of our final sample size and will acknowledge the possibility of not detecting a difference in comparisons where we lack adequate power. |
| 1. **Data Linkage Required (if applicable):^§^**   **^§^***Please note that the data linkage/s requested in research protocols will be published by the CPRD as part of its transparency policy*  We require data from CPRD Gold, HES admitted patient care, ONS Death Registration Data, and Patient level index of multiple deprivation. HES and ONS data are required to ascertain specified outcomes – hospitalisation due to UTI, sepsis, pyelonephritis or acute kidney injury, and death. Although these outcomes may be ascertainable from primary care records, previous work has shown that event capture is more accurate with linked data, compared with single data sources.^13^ Furthermore, ONS data is essential to our second objective related to describing causes of early death. Deprivation quintile is a specified confounder variable. |
| 1. **Study population**   Inclusion criteria:  Patients are eligible for inclusion if, between 1^st^ Jan 2010 and 31^st^ December 2016:  Their data is deemed as ‘up-to-standard’ and flagged as ‘acceptable’ by CPRD,  They are ≥65 years old,  Their practice has consented to data-linkage,  They have a record of an incident empirically treated UTI in their primary care data. We define this as the **first** primary care record of a relevant UTI related Read code within the study period (code list in Appendix), with a same-day antibiotic prescription, with no record attributable to a probable UTI event in the previous 90 days, and no hospital discharge in the previous 14 days (to guard against including hospital acquired UTIs).  Exclusion criteria:  We will exclude patients if they are temporary residents or have gaps in their data coverage.  Follow-up begins on the day of the incident UTI. Follow-up ends on the day of a record indicating any of the specified outcomes, or at the earliest of, 28 days following the incident UTI, study end date (31^st^ December 2016), or last day of available CPRD data. |
| 1. **Selection of comparison group(s) or controls**   To compare outcomes for different antibiotics, the cohort will include all adults aged ≥65 with an incident episode of empirically treated community acquired UTI. To investigate antibiotic choice, we will estimate risk of each outcome using nitrofurantoin as the comparator. To investigate antibiotic duration, we will estimate risk of each outcome using the duration recommended by clinical guidelines as the comparator, i.e., 3 days in women and 7 days in men. Thus, all estimates will be gender-specific. For the propensity score matched analyses, patients receiving the reference treatment (e.g., nitrofurantoin), will be matched by age and propensity score to patients receiving an alternative treatment.  To compare outcomes by renal function, we will categorise function according to estimated glomerular filtration rate (eGFR) as per the categories used by NICE. We will ascertain eGFRs using data recorded in the “test” table in the 24 months prior to the incident UTI, or calculate eGFR from a record of creatinine (“test” table) in the prior 24 months, plus age, gender and ethnicity. We will estimate risk of each outcome using those with an eGFR of ≥60 ml/min/1.73m^2^ as the comparator. Secondly, we will estimate risk of each outcome in each eGFR category, comparing nitrofurantoin with trimethoprim, using age, gender and propensity score to match those prescribed nitrofurantoin with those prescribed trimethoprim. |
| 1. **Exposures, Health Outcomes^§^ and Covariates**   **^§^***Please note:* *Summary information on health outcomes (as included on the ISAC application form above )will be published on CPRD’s website as part of its transparency policy*  **To estimate risk of each outcome according to antibiotic choice:**  **Exposure**  Acute antibiotic prescription  **Outcomes**   1. Primary care re-consultation: a primary care record of urinary tract symptoms or UTI related diagnoses with/without a same-day antibiotic prescription within 14 days of the incident UTI. 2. Primary care antibiotic prescription: any primary care record of an acute antibiotic prescription within 14 days of the incident UTI. 3. Hospitalisation for UTI, pyelonephritis, sepsis or acute kidney injury: ascertained from the “HES_diagnosis_epi” table using relevant ICD-10 codes (code list in appendix) assigned to the first episode of the first hospital spell within 14 days of the incident UTI. 4. Death within 28 days of the incident UTI: ascertained using linked ONS death registry data.   **Covariates**  We will adjust for covariates in a multivariable regression analysis, and use covariates to compute a propensity-score in the matched analysis. We will define covariates using data inputted prior to the date of the incident UTI. We will include the following covariates:  Age in years,  Year of incident UTI,  Index of multiple deprivation quintile,  Based on relevant medcodes from the ‘clinical’ table, the presence or absence of:  diabetes,  dementia,  coronary heart disease,  renal disease,  stroke,  cancer  metastatic cancer,  heart failure,  urinary incontinence.  Presence or absence of a catheter in the previous 12 months using the “medcode” and “prodcode” variables.  Charlson score using a previously published list of Read codes ^14^ and operationalised for our previous analyses in protocol 15_027A,  Whether housebound or not,  Whether resident in a care home  Polypharmacy (defined as >=5 repeat medications) using mean number of medications on repeat prescription during the previous 12 months,  Estimated glomerular filtration rate in the previous 12 months,  Duration of antibiotic prescription  **To estimate risk of each outcomes according to antibiotic duration:**  **Exposure**  Duration of acute antibiotic prescription – categorised as ≤3 days, 3-6 days, 7 days, >7 days. Calculated using the “ndd” and “qty” variables in the “Therapy” table, as per in our previous analysis for protocol (15_027A).  **Outcomes**  As above  **Covariates**  As above but replacing antibiotic duration with antibiotic type.  **To estimate risk of each outcomes according to renal function:**  **Exposure:**  eGFR categorised into 4 groups (≥60 ml/min/1.73m^2^, 45-59 ml/min/1,73m^2^, 30-44 ml/min/1,73m^2^, <30 ml/min/1,73m^2^.  **Outcomes**  As above  **Covariates**  As above but not including eGFR/renal disease. |
| 1. **Data/ Statistical Analysis**   We will report the number of adults ≥65 in the database each year between 2010 and 2016 and estimate annual rates/proportions and 95% confidence intervals for those presenting with suspected UTI and those prescribed empirical antibiotics. We will use appropriate summary statistics and uncertainty measures to describe antibiotic choice and duration.  To compare outcomes by antibiotic choice and duration, we will use appropriate summary statistics (means and standard deviations for continuous variables and rates/proportions for categorical variables) to allow comparison of baseline characteristics of patients in each treatment group, both for our multivariable regression analyses and our propensity-score matched analyses. We will use multivariable logistic regression to compute propensity scores for the probability of receiving nitrofurantoin, and the probability of receiving guideline adherent therapy duration, using all covariates listed in section (M) as predictors, except age and gender which will be matched outside the model. Therefore, patients will be matched according to age (+/- 1 year), gender, and propensity score. We will accept an absolute standardised difference of <10% on all covariates as adequate balance. We will compare outcomes for different treatment strategies in patients with similar propensity scores. We will use multivariable logistic regression to estimate odds of each outcome for each comparison.  We will use multivariable logistic regression to estimate odds of each outcome for patients with differing renal function prescribed empirical nitrofurantoin. We will use eGFR as the predictor variable. We will then compute propensity scores for the probability of empirical nitrofurantoin prescribing in those with reduced eGFR and compare outcomes with those of similar propensity score prescribed empirical trimethoprim.  Finally, we will use appropriate summary statistics to describe causes of any hospitalisation or death within 7 days of the incident UTI and use logistic regression to identify any clinically meaningful predictors.  We will regard a p<0.05 as statistically significant and an effect size of >10% as clinically significant. |
| 1. **Plan for addressing confounding**   We will address confounding by adjusting for important confounders in the multivariable regression model, repeating the analyses using a propensity-score matched approach, and by checking for consistency of effect in sensitivity analyses. However, residual confounding may still occur through unmeasured confounders and confounding by indication. We will therefore highlight these issues when we interpret and report our findings. |
| 1. **Plans for addressing missing data**   We do not anticipate missing data to impact upon outcome ascertainment. If there are missing data in the covariates/subgroups (e.g., for estimated glomerular filtration rate), we will consider using multiple imputation by chained equations. Based on our previous analyses, we expect eGFR data to be missing for about 10-15% of our cohort. |
| 1. **Patient or user group involvement (if applicable)**   The proposed research forms part of a wider program of work funded by an NIHR Doctoral Research Fellowship and supported by a lay advisory panel. This panel have contributed to the program of work by attending project management meetings and contributing to overall study direction. For this specific research project, the lay advisory panel contributed to the study objectives and helped to define key outcomes and their importance to patients and their families. |
| 1. **Plans for disseminating and communicating study results, including the presence or absence of any restrictions on the extent and timing of publication**   Dissemination will occur through relevant conferences, peer-reviewed journals and through lay summaries for relevant patient groups as per advice from our lay advisory panel. |
| 1. **Limitations of the study design, data sources, and analytic methods**   Limitations are:   1. We do not have microbiological data and thus, will be unable to comment on relationships between microbiologically confirmed UTI and outcomes. 2. Clinical codes are subject to difference in coding behaviours between clinicians and thus some UTIs will be missed (where no codes or non-specific codes have been used), and some UTIs will be misclassified. We will estimate the proportion of UTIs we may have missed by calculating the proportion of prescriptions for nitrofurantoin and trimethoprim (used almost exclusively for UTI in the UK) without one of our inclusion codes. We will reduce misclassification to a degree, as we are selecting cases on clinical code and same-day antibiotic, but will be aware of atypical antibiotics. 3. Despite the analysis plan outlined above, there is still a chance that any observed difference between the two groups could be due to confounding, in particular, confounding by indication. |
| 1. **References**   1. McDonald HI, Nitsch D, Millett ER, et al. New estimates of the burden of acute community-acquired infections among older people with diabetes mellitus: a retrospective cohort study using linked electronic health records. *Diabet Med* 2014;31(5):606-14. doi: 10.1111/dme.12384 [published Online First: 2013/12/18]  2. Drekonja DM, Rector TS, Cutting A, et al. Urinary tract infection in male veterans: treatment patterns and outcomes. *JAMA Intern Med* 2013;173(1):62-8. doi: 10.1001/2013.jamainternmed.829 [published Online First: 2012/12/06]  3. Knottnerus BJ, Grigoryan L, Geerlings SE, et al. Comparative effectiveness of antibiotics for uncomplicated urinary tract infections: network meta-analysis of randomized trials. *Fam Pract* 2012;29(6):659-70. doi: 10.1093/fampra/cms029 [published Online First: 2012/04/21]  4. Huttner A, Verhaegh EM, Harbarth S, et al. Nitrofurantoin revisited: a systematic review and meta-analysis of controlled trials. *J Antimicrob Chemother* 2015;70(9):2456-64. doi: 10.1093/jac/dkv147 [published Online First: 2015/06/13]  5. Bjerrum L, Dessau RB, Hallas J. Treatment failures after antibiotic therapy of uncomplicated urinary tract infections. A prescription database study. *Scand J Prim Health Care* 2002;20(2):97-101. [published Online First: 2002/08/20]  6. Lee MTG, Lee SH, Chang SS, et al. Comparative Effectiveness of Different Oral Antibiotics Regimens for Treatment of Urinary Tract Infection in Outpatients: An Analysis of National Representative Claims Database. *Medicine (Baltimore)* 2014;93(28) doi: 10.1097/md.0000000000000304  7. Lutters M, Vogt-Ferrier NB. Antibiotic duration for treating uncomplicated, symptomatic lower urinary tract infections in elderly women. *Cochrane Database Syst Rev* 2008(3):Cd001535. doi: 10.1002/14651858.CD001535.pub2 [published Online First: 2008/07/23]  8. Hawker JI, Smith S, Smith GE, et al. Trends in antibiotic prescribing in primary care for clinical syndromes subject to national recommendations to reduce antibiotic resistance, UK 1995–2011: analysis of a large database of primary care consultations. 2014 doi: 10.1093/jac/dku291  9. SIGN. Management of suspected bacterial urinary tract infection in adults: Scottish Intercollegiate Guidelines Network; 2015 [Available from: <http://www.sign.ac.uk/pdf/sign88.pdf>.  10. Oplinger M, Andrews CO. Nitrofurantoin contraindication in patients with a creatinine clearance below 60 mL/min: looking for the evidence. *Ann Pharmacother* 2013;47(1):106-11. doi: 10.1345/aph.1R352 [published Online First: 2013/01/24]  11. Geerts AF, Eppenga WL, Heerdink R, et al. Ineffectiveness and adverse events of nitrofurantoin in women with urinary tract infection and renal impairment in primary care. *Eur J Clin Pharmacol* 2013;69(9):1701-7. doi: 10.1007/s00228-013-1520-x [published Online First: 2013/05/11]  12. Singh N, Gandhi S, McArthur E, et al. Kidney function and the use of nitrofurantoin to treat urinary tract infections in older women. *Cmaj* 2015;187(9):648-56. doi: 10.1503/cmaj.150067 [published Online First: 2015/04/29]  13. Millett ER, Quint JK, De Stavola BL, et al. Improved incidence estimates from linked vs. stand-alone electronic health records. *J Clin Epidemiol* 2016;75:66-9. doi: 10.1016/j.jclinepi.2016.01.005 [published Online First: 2016/01/19]  14. Khan NF, Perera R, Harper S, et al. Adaptation and validation of the Charlson Index for Read/OXMIS coded databases. *BMC Family Practice* 2010;11(1):1. doi: info:pmid/20051110 |
